# Towards Customer-Centric Additive Manufacturing: Making Human-Centered 3D Design Tools through a Handheld-Based Multi-Touch User Interface †

**DOI:** 10.3390/s20154255

**Published:** 2020-07-30

**Authors:** Ivan Rodriguez-Conde, Celso Campos

**Affiliations:** 1 Department of Computer Science, University of Arkansas at Little Rock, 2801 South University Avenue, Little Rock, AK 72204, USA; 2 Department of Computer Science, Campus As Lagoas, University of Vigo, 32004 Ourense, Spain; ccampos@uvigo.es

**Keywords:** Industry 4.0, customer-centric manufacturing, computer-aided design, multi-touch interaction, human-centered computing, handheld devices

## Abstract

Seeking a more flexible and efficient production, additive manufacturing (AM) has emerged as a major player in the industrial field, streamlining the fabrication of custom tangible assets by directly 3D printing them. However, production still takes too long due to printing, but also due to the product design stage, in which the customer works together with an expert to create a 3D model of the targeted product by means of computer-aided design (CAD) software. Skipping intermediate agents and making customers responsible for the design process will reduce waiting times and speed up the manufacturing process. This work is conceived as a first step towards that optimized AM model, being aimed at bringing CAD tools closer to clients through an enhanced user experience, and consequently at simplifying pre-manufacturing design tasks. Specifically, as an alternative to the traditional user interface operated with the keyboard and mouse duo, standard in CAD and AM, the paper presents a comprehensive multi-touch interaction system conceived as a customer-centric human-machine interface. To depict the proposed solutions, we adopt furniture manufacturing as a case study and, supported by a CAD-like software prototype for 3D modeling of custom cabinets introduced in a previous work of the authors, we assess our approach’s validity in terms of usability by conducting in-lab and remote user studies. The comparison between the designed multi-touch interaction and its desktop alternative yields promising results, showing improved performance and higher satisfaction of the end-user for the touch-based approach, that lay the groundwork for a smarter factory vision based on remotely-operated AM.

## 1. Introduction

During the last decade, both progress in telecommunications and the ever-greater miniaturization of electronics have led to the emergence of innovations such as the internet of things (IoT) and cyber-physical systems (CPS), able to incorporate computing, communication, and even “smart” features, until recently only available in traditional computers, to ordinary objects such as appliances or cars. This technological revolution has had a tremendous effect, and terms such as smart home, smart car, and smart city are today part of the everyday vocabulary of millions of people no matter what their IT background is [1]. The global impact goes beyond people’s personal or domestic realm. The integration of IoT and CPS in the industrial world, driven by the progressive adoption of advances in fields such as mobile internet, cloud computing, big data, machine-to-machine communication, data analysis, or artificial intelligence, is rapidly transforming traditional manufacturing processes, leading to what has been named, depending on the author and the context, as Industry 4.0 (I4.0) [2,3], industrial internet of things [4], cyber-physical manufacturing systems [5], or smart manufacturing [6,7].

The aforementioned technological innovations are the main driving force behind the fourth industrial revolution already underway. Research and progress in production methods, materials, and product design aim to create a more robust, competitive, and flexible industry. They seek to improve production efficiency to satisfy the traditional market demands for high-quality and low-cost products, as well as to achieve higher customer satisfaction by providing highly customized services and products without requiring longer waiting time for the customer, nor longer manufacturing time [8]. Such reduction in production time, and the consequent improvement of the manufacturing process, is one of the main challenges researchers and technologists will have to address in the coming years on the way to the pursued I4.0.

In this I4.0 context, focused on the search for a more efficient and flexible production, additive manufacturing (AM) has emerged as an alternative of great interest in multiple fields, both in the commercial and research domains [9,10]. This manufacturing paradigm, also known as 3D printing and direct digital manufacturing, consists of the fabrication of tangible assets directly from their digital 3D models. This: (i) enables greater freedom and flexibility in product design, (ii) facilitates the evaluation and comparison of different ideas or solutions, and (iii) allows better tailoring of the final design to customer-specific requirements [9,10]. In short, it makes it possible to manufacture complex and customized products with low production volumes and frequent design changes. However, despite the above, its presence in the industrial environment remains limited due to problems such as low precision and high production times, especially when compared to traditional production processes such as those based on computer numerical control machines [9]. Such longer production time stems not only from the 3D printing time but also from the time spent on the design stage.

Though customers are ultimately responsible for deciding on the final configuration of the product, they usually lack the expertise to approach the design process on their own. Thus, as a rule, in that pre-production stage, individuals are comprehensively supported by the manufacturing personnel, both working together, typically asynchronously, to create an initial prototype. Such traditional model means unnecessary waiting time for the customer and an increasing cost of product manufacturing. Simplifying tasks will bring the design process closer to the customer, allowing him to bypass the support provided by third parties and consequently enabling an enhanced customer-centric manufacturing workflow. Streamlining the design process, however, is not trivial. 3D modeling applications, in general, and computer-aided design (CAD) tools, in particular, can provide technological support for design tasks, common in the day-to-day work of many engineering professionals [11,12,13]. Their purpose is essentially replacing traditional manual paper-based sketching by a semi-automated process, thus facilitating a theoretically more agile [14] conceptual design of real-world objects. However, though modern hardware is inexpensive and powerful enough to allow the end-users to approach 3D modeling, the complexity of these tasks and, consequently, of the supporting software tools is still significant.

Such complexity and the subsequent reluctance of conventional users to adopt this type of software solutions result from the underlying philosophy. In general, they primarily seek to model real-world objects as faithfully as possible, regardless of the specific users’ needs, and mainly at the expense of their experience. We are talking about tools that provide a wide range of functionalities presented from a windows, icons, menus, and pointing (WIMP) interface [15] typically consisting of excessively populated menus and toolbars. That makes the mere identification of a specific control (that is, finding its location in the interface) more complex in those systems than in other types of tools, resulting in a higher cognitive load and learning time.

Apart from that, the WIMP interface, while usually considered to be a synonym of direct manipulation [16], may not be a significant metaphor in specific three-dimensional-based design use cases. Specifically, in real-life contexts, individuals can directly manipulate both modeled objects and related design tools, such as pencils and pens, when performing conceptual design and modeling tasks. However, in CAD-based environments, users are commonly required to use traditional input devices (keyboard and mouse) in order to work with the different widgets on the graphical user interface (GUI) and edit the constituent elements of the design. Thus, it is clear that the experience provided by such tools is far from real modeling practices; so, consequently, we can assume that the supporting interaction is not very natural or intuitive.

To the extent we can fill the gap described, we will succeed in bringing the creative process closer to the general public, as well as in bringing their human decision-making capabilities inside the control loop of AM systems. The search for solutions must be aimed at simplifying design tasks and improving the user-system interaction to allow individuals to perform CAD-specific modeling jobs autonomously. In this context, we propose as a possible approach an interaction model based on a multi-touch interface, recognized in human-computer interaction (HCI) as one of the most direct interaction techniques [17]. We provide a global interaction solution, contributing novel solutions to problems traditionally associated with the touch interface. Supported by a CAD-like software prototype for 3D modeling of custom cabinets introduced in previous work [18,19], we also explore the value of the designed mechanisms by conducting two different studies with real users to analyze and determine to what extent the conceived touch-based human-machine communication style affords a proper experience in terms of usability for low-skilled users and, ultimately, to get a sense of the feasibility and potential usefulness of a multi-touch human-machine interface (HMI) for CAD tasks in the AM context.

**Figure 1:**
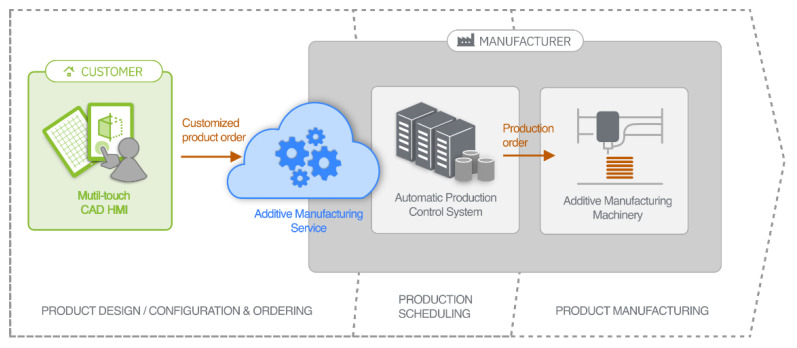
Realization of the customer-centric additive manufacturing vision based on the Multi-touch (computer-aided design human-machine interface) CAD HMI.

An HMI like the one pursued, conceived on the basis of the multi-touch user interaction supported by current handheld mobile devices, would provide customers with an intuitive tool for modeling and customizing the goods to be produced, but would also constitute a gateway and remote control of a potential distributed manufacturing service in which cloud technologies would undoubtedly play an important role. As there would be no intervention by the manufacturer’s staff, having finished the design, the customer would be the one to send through the HMI the order to the automatic production control system (APCS), accessible in the form of a cloud-based service. The APCS would then handle the incoming remote product orders and, based on data regarding performance, material availability or work in progress, dispatch production jobs to the available or most suitable 3D printing machine to keep the manufacturing cycle time as low as possible. Figure 1 provides a diagram that depicts the different constituent elements of the notional remotely-controlled AM solution just described.

## 2. Related Work

### 2.1. Human-Machine Interaction in Industry 4.0

As indicated in the previous section, the so-called Industry 4.0 has arisen on the basis of the traditional industry, thanks to the adoption and application of solutions and paradigms from the Information and Communication Technology field. It represents the vision of an interconnected and automatized industry that may seem to depict a scenario in which, a priori, human workers would be potentially replaced by smarter machinery components or entities capable of making decisions individually as well as communicating and coordinating themselves in order to achieve production goals autonomously [20]. However, some current trends in I4.0 reject that hypothesis of a dehumanized coming industry, focusing on operators and their role as agents able to adapt to a changing work environment and perform a wide range of different jobs within production systems [21].

In the context of mass customization and flexible manufacturing, operators are seen as key entities in next-generation productive systems. To emerge as the center of that new industry, they will have to abandon their current role of merely specialized executors of mechanical and repetitive tasks, and become an active part of the system, capable of performing a wide range of different physical and cognitive tasks [22,23]. Still, given the more complex nature of CPS, training in the underlying technological paradigms and tools will be critical to enable individuals to interact with their work environment more efficiently [23] and, therefore, accomplish the indicated transition. With that in mind, integrating human personnel with the hardware and software infrastructure is shown to be one of the primary requirements to be met in CPSs [24,25,26], being of vital importance the exploration of interaction mechanisms for a better man-machine communication [23,27].

In recent years extensive research on UIs has been conducted pursuing the design and development of new HMIs in I4.0 [28]. UIs are understood as a core component to facilitate machinery support in order to increase the efficiency, and, in general, enhance the capacities of human personnel when performing tasks such as planning [29] and product design [30], quality control [31], or maintenance [24], being commonly used, for instance, to transmit instructions to operators, provide navigation services [32], or to enable operators to control industrial robots [33]. Hence, generally speaking, it is possible to state that UI-specific works present in the related literature have been mainly aimed at improving and expanding the operators’ working environment. Only a few merely testimonial studies such as [34,35] have been focused on providing added value to other actors such as end customers.

Regarding the HMI landscape in I4.0, there is some agreement among the authors in identifying virtual reality (VR) [36,37], and augmented reality (AR) [36,38] as the two main technological drivers of the new generation of interfaces [23,27,39]. Those technologies allow users to operate in complex 3D workspaces, providing an immersive and realistic visualization as well as a natural interaction [40] through multiple sensorial channels (e.g., vision, sound, touch, smell, taste). They constitute a hardware/software infrastructure especially suitable for industrial research and development practices such as rapid prototyping of both products and manufacturing equipment [41], and also for training of personnel pursuing a more efficient [42] and safe [43] human-machine interaction. Furthermore, AR in particular, unlike VR, does not replace the real world with a synthetic one. It seeks to improve the experience of individuals in the real world by superimposing computer-generated perceptual information on it [29] through a ubiquitous and pseudo-transparent interface, allowing a contextualized visualization of complex data [44] and, consequently, providing valuable support for tasks such as maintenance, repair, assembly or creation of virtual content [38,45].

Immersive technologies have shown a considerable potential to transform both production processes and customer experience, and even to reshape the internal organization of manufacturing companies comprehensively. However, despite this and the significant number of AR and VR applications already existing in the industrial field, their expansion among the general public is still limited [46]. For this reason, they do not largely constitute an appropriate response to designing end-customer-oriented software solutions yet, and, consequently, it seems reasonable to assume that the search space for the latter should be centered on innovative technologies and interaction metaphors already established in the consumer goods market. Recent studies advocate current mobile devices as the third primary HMI alternative in I4.0 [23]. Today’s mobile devices’ connectivity and sensing capabilities enable the convergence of the operational possibilities of multi-touch screens, voice recognition, and gesture recognition, as well as remote access to services of potential interest, providing the user with a natural, multimodal and ubiquitous interface. The adoption and application of such interaction style for manufacturing processes allow component manufacturers and system developers greater customization [44], and ease of use [47].

### 2.2. Finger-Based Interaction with Touch Handheld Devices

The proliferation of mobile devices and the democratization of multi-touch technology have led to a leap from graphic interfaces, as we knew them, to touch-based interfaces. However, despite its relatively recent commercial interest, multi-touch technology has been widely studied in the scientific field over the last thirty years. Many authors have focused on exploring and taking advantage of direct interaction styles, resulting in a plethora of touch-based and gesture-driven techniques. The possibility of touching data and manipulating them directly on the screen simplifies the interaction process [17,48]. It also makes it possible to adapt real-world interactions to the digital environment, improving individual’s intuition [48] and, consequently, favoring less IT skilled users [17,49].

However, the mere use of fingers as pointing and selection tools does not guarantee improved performance and end-user satisfaction. The experience may be degraded not only by limitations of the supporting hardware, such as the absence of haptic feedback [50] or the complexity of textual data entry [51,52,53] but also by specific issues of the interaction style, such as the complexity intrinsic to the use of symbolic gestures [54,55] or the ambiguity and lack of accuracy [17,56].

In particular, ambiguity and lack of accuracy have received the most attention in the touch-interaction-related corpus due to their undoubted negative impact on finger-based pointing and selection operations. More specifically, finger’s size and opacity together with skin’s elasticity generate the occlusion of a portion of the screen, potentially causing partial or total hiding of on-screen elements [17], and also discrepancy between the exact location of touched spots and the user’s expectation [56]. To address those problems, we find in the scientific literature several approaches aimed at obtaining a more accurate touch interaction.

#### 2.2.1. Increasing the Size of On-Screen Widgets

Pioneering works such as Sears and Schneiderman’s [57] and more recent studies, such as the one conducted by Wang and Ren [58], revealed the minimum dimensions that every on-screen widget must have to make target selection reliable. Reported values represent, however, an ideal that, in many cases, may not be feasible, given the design requirements and spatial constraints imposed by the physical size of touch surfaces. In this context, several authors have tried to improve target acquisition by temporarily and selectively modifying scale values. Parker et al. [59] grouped those techniques into three distinct approaches: (*i*) expanding the target, based on the use of dynamically-sized graphic elements [60,61]; (*ii*) using area cursors [62], that is, cursors capable of dynamically adapting their size depending on the proximity of the surrounding targets [63]; (*iii*) semantic targeting, a solution devised by Blanch et al. [64] which involves expanding targets only in motor space and in correlation to their proximity to the cursor.

#### 2.2.2. Disambiguation Movements

Increasing the size of on-screen elements, in spite of improving user performance in pointing and selection operations, usually causes an unwanted ambiguity in heavily-populated environments. Techniques such as Escape [65] were presented as a possible solution to this problem, requiring the user to perform successive corrective dragging movements to firstly determine which specific target to select and then finalize its acquisition through a selection mechanism such as Take-off [66] or DTMouse [67]. Thus, users must drag a finger on the interaction surface in the direction of the desired target and make an additional confirmation gesture such as lifting the finger [66]. That increases the complexity of the gesture, and consequently, it slows down the interaction and makes it more uncomfortable.

#### 2.2.3. Cursor Offset

This refers to a collection of techniques, explicitly designed for cursor-based touch systems, that seek to avoid the occlusion of both the cursor and the selectable targets by applying an offset to the pointer: the pointing widget is moved away from the finger’s contact surface and thus placed in a visible area of the screen. Inspired by the offset cursor method introduced by Potter et al. [66], solutions such as virtual keys and cross-keys [17], dual finger offset and dual finger midpoint [49], or Magstick [68] have proven to be an effective way of addressing the occlusion problem. However, they also cause other significant interaction difficulties, increasing the cognitive effort and the time required to carry out selection tasks, and even preventing the selection of some targets.

#### 2.2.4. Call-Outs

Like the previous approach, it constitutes an attempt to address the occlusion problem, being its most representative method, the Shift technique introduced by Vogel and Baudish [69]. That technique considers the creation of additional display areas or call-outs every time the user touches the screen. Through those call-outs, individuals are provided with visual feedback about the exact location of the touchpoint in the occluded area. More recent techniques such as Thumbspace [70] and occlusion-aware interfaces [71] follow the main principles defined by Shift and seek to facilitate target acquisition by providing additional on-screen interaction and display areas. In particular, Thumbspace proposes the use of a software touchpad that overlays the rest of the on-screen elements to enable users to interact with the GUI by using the thumb; while the occlusion-aware interfaces are designed to detect occluded areas and, in response, provide the corresponding visualization through a bubble call-out.

As can be seen, the design of a successful touch interface can be challenging despite its higher interactive richness. In that sense, it is easy to foresee that the use of this type of interface in CAD systems, in which tasks are primarily based on pointing and selection operations, is not trivial and will demand the exploration and design of solutions to solve, or at least minimize, the negative impact of the mentioned interaction-related issues.

## 3. Touch-Based Interaction System

As stated in the first point of the paper, the ultimate goal of the present work is to streamline the fabrication of goods with AM systems. We seek to optimize the standard manufacturing workflow with such systems, minimizing pre-production time by simplifying design tasks, mainly consisting of modeling operations typically performed by means of CAD software. Our approach puts customers at the center of the design process, facilitating the use of CAD tools by any individual regardless of his/her expertise using computer applications, and, thus, enabling them to perform design operations with no need for third party support. Based on the multi-touch capabilities of today’s mobile hardware, we propose a comprehensive interaction solution that combines the use of a GUI with the use of a gesture-based natural interface to smooth the steeped learning curve commonly associated to CAD software. In particular, the conceived interaction system is the result of three design principles:

- the minimization of the GUI,
- the creation of a touch-based interface for the direct manipulation of design elements,
- the minimization and simplification of mode switching.

Throughout this section, we provide a more in-depth look at the specific conceived interaction mechanisms that stemmed from each of the design principles mentioned above and at the different solutions proposed to address interaction issues traditionally associated with the use of fingers in target selection operations. Furthermore, since the conception of UIs is closely linked to the specific tasks it supports, we contextualize the contributions in a specific case study – specifically, custom furniture manufacturing—to support and depict the mechanisms and techniques designed.

### 3.1. Case Study: Digital Prototyping of Custom Cabinets

The design process supported by CAD software, though it depends to a certain extent on the particular features of the specific software applications used, encompasses a number of features or operations that can intuitively be identified as common in CAD environments, such as the addition of new elements to a digital 3D model, the application on it of basic scaling and translation transformations, or the free exploration of the 3D scene through interactive camera manipulation.

Contextualized in the design of custom cabinets, the set case study is supported by a specific workflow but perfectly exemplifies the main tasks carried out by the user in the use of CAD tools. Specifically, the workflow begins with the creation of a floor plan of the room where the cabinet is supposed to be installed. Next, the external frame of the cabinet is created taking one of the walls in the plan as reference. Subsequently, throughout an iterative process of division of the internal space, its final structure is progressively outlined. Additionally, to complete the internal layout, the related accessories are also included; and, finally, the sketch is finished by incorporating the front doors.

#### 3.1.1. Room Edition

The primary aim of this module is to obtain a geometric model of the external structure of the cabinet from a basic blueprint of the room. The user defines in the first place that blueprint by creating the walls and pillars that make up the structural outline of the room. The position, orientation and size of the cabinet are then calculated in an almost direct way, taking the geometry of the selected supporting wall as the reference. To do this: (*i*) The origin of the cabinet is translated to the position of the origin point of the wall; (*ii*) the cabinet is rotated and oriented towards the direction of the normal vector of the wall; and (*iii*) the width and height of the cabinet are both derived from the analogous dimensions of the wall.

#### 3.1.2. Interior Layout Edition

**Figure 2:**
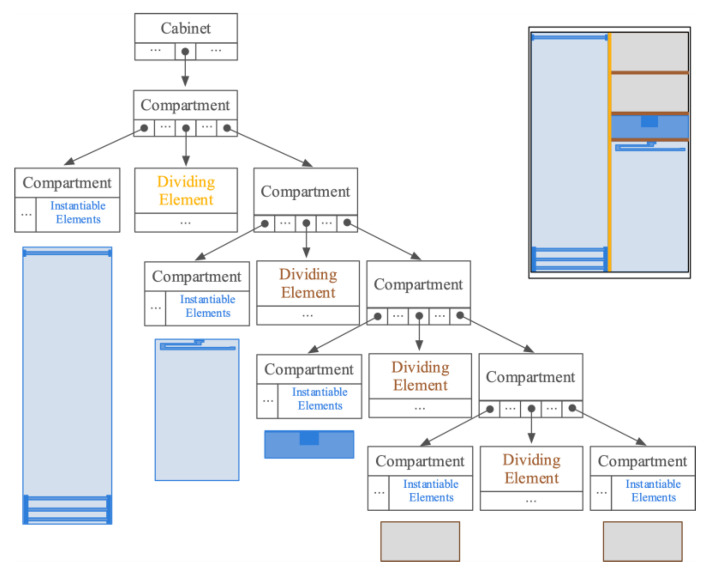
Diagram representing the *Cabinet* structure for a two-body cabinet.

From the base model created in the previous stage, users can refine the design by incorporating additional geometric elements representative of the internal structure of the cabinet. The topological and geometrical information of these elements is formalized and managed internally by means of a Cabinet data structure, conceived as a tree (See Figure 2).

The addition of a horizontal or vertical dividing element to an empty spatial unit or compartment results in the creation of new tree nodes. Each spatial unit keeps all the references of the several dividing elements and the resulting child spatial units within. Every dividing element, for its part, is geometrically represented by a unit cube, rotated based on the orientation of the whole cabinet, and scaled and positioned based on the local coordinates system of their enclosing space. Compartments that do not contain any dividing element are considered terminal nodes and keep references of instantiable elements representative of the several accessories attached.

In the same spatial unit, it is possible to find different types of accessories (for instance, drawers, bars, or shoe racks) but also several occurrences of the same hardware. The structure and, consequently, the geometry of those accessories can be complex, making their processing and visualization time and resource consuming. We alleviate this situation with an algorithmic solution named as *instantiation* that generates the visual representation of the accessories more efficiently, storing the geometric data of each one just once, and using a reference to that information in order to showcase each new occurrence, a replica object that differs from the original model in size, position or orientation.

#### 3.1.3. Door Edition

In the final step of the sketching process, the end-user incorporates the front doors to the design. Each door is represented by a data structure named *Door*, analogous to the *Cabinet* data structure introduced in the previous point. Thus, each *Door* is internally defined as a tree that keeps data regarding two different types of constituent elements, both geometrically characterized as unit cubes: an entity type named *Panel* that represents the several stretches that make up the surface of every door; and, additionally, an entity called *Division* that represents each existing horizontal division between panels.

Supported by the Door data structure, the creation of each door is conceived as a three-step sequential process:

1. The creation of a starter geometrical model. Based on a mechanism transparent to the user, the system autonomously creates a model of a plain (undivided) door, computing the scale, translation and rotation factors needed to adequately accommodate it to the rest of the elements of the cabinet.
2. The division of the initial model into panels. The transformed cube resulted from the previous step is segmented into several cubes that represent both panels and divisions. The underlying mechanism in this stage requires user intervention to define the number and position of divisions added to the door.
3. The configuration of the visual appearance. Users can define a material or color for each panel in the door. The front face of the related cube is textured with the corresponding bitmap in order to give the panel model the desired visual finish.

### 3.2. Minimization of the Graphic User Interface

**Figure 3:**
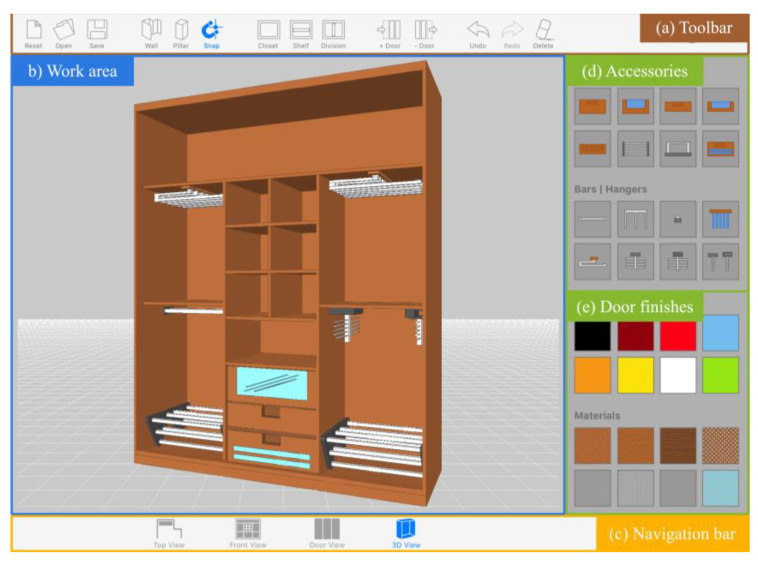
(graphical user interface) GUI layout of the multi-touch prototype, designed based on a canvas metaphor, and consisting of (**a**) central work area where the user builds the cabinet design, (**b**) a top toolbar featuring the available cabinet creation tools, (**c**) a bottom navigation bar allowing the user to toggle between the different module-specific viewpoints, and a side panel that provides direct access to a set of accessories (**d**) that can be included inside the cabinet to refine the design, as well as the materials (**e**) that can be used for the door front panels.

We exclude non-primary on-screen controls to design a GUI far from the visual clutter typical in WIMP-based applications. This approach seeks to minimize the lack of precision and ambiguity problems caused by “overcrowded” interfaces in touch-based systems [17,57] and thus avoid the consequent negative impact on the performance and satisfaction of the end-user. As showcased in Figure 3, our proposal is based on a canvas metaphor: we get rid of superfluous elements to present a clean and minimalist GUI, with a clear leading role of an interactive 3D viewer, conceived as design slate for a more immersive and natural experience.

The interactive 3D viewer (Figure 3b) represents the work area where users perform the different design tasks. From that central panel, users directly manipulate the design of the cabinet, and, in response, the system provides constant feedback, displaying in real time the results obtained from the creation or editing operations. The content provided from the slate is dynamic and changes throughout the design flow. For each functional module, the 3D viewer renders the relevant specific objects, thus providing the appropriate visualization in each case. For example, the room edition module provides a zenithal view of the several walls and pillars of the room, while the interior *layout edition* module provides a front view with just the elements included within the cabinet, omitting both doors and surrounding walls.

**Figure 4:**
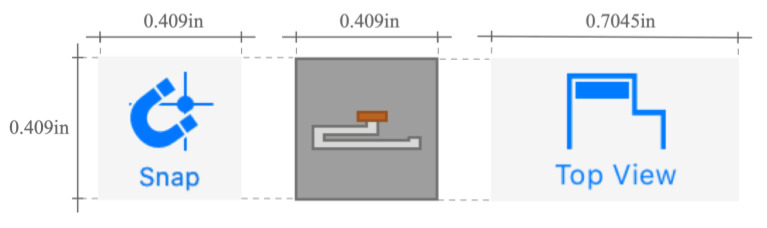
Several button types included in the GUI of the conceived multi-touch System and their corresponding sizes.

Along with the 3D viewer, the graphic interface features a set of GUI controls that provide the users with access to the available functionalities. Regarding those controls, our efforts have been focused on selecting the most appropriate type and size to support finger-touch interaction successfully. With that in mind:

- Standard widgets such as menu bars or drop-down lists, which usually force the user to navigate between different depth levels to access a particular option, were discarded. The user can access every tool o functionality through simple buttons that can be tapped directly and in a fast way.
- Buttons were given an appropriate size to accommodate tap events to finger-based operations, according to the studies [57,58] cited in the related works section. Specifically, the conventions derived from those studies establish as the minimum physical size of the selectable targets a value between 10.5 and 26 mm per side (0.409 and 1.02 inches respectively). Figure 4 shows the specific values finally used for each button type included in the graphic interface.

### 3.3. Multi-Touch Interface for the Direct Manipulation of Design Elements

On the basis of the described graphic interface, we conceived a multi-touch interaction model that allows users to operate with their fingers directly on any GUI control and design element presented from the 3D viewer. In particular, user-system communication is mostly channeled through the central drawing canvas: the interaction with GUI controls is formalized as simple discrete touches, while the interaction with the design slate is modeled as a set of operations performed consistently throughout the design process via recurrent touches and symbolic gestures.

#### 3.3.1. Action Patterns

In terms of cabinet model manipulation, it is considered that the first touch on the interaction surface is performed by the index finger of the dominant hand (named primary finger), and that every additional touch is made by any other finger from either hand. Individuals use their primary finger as a pointing device to create and define the different structural and visual features of the cabinet, in a communication exercise common to all tasks involved in the design process. We define four distinct action patterns to support those tasks.

##### Single or Multiple Taps on a GUI Control

**Figure 5:**
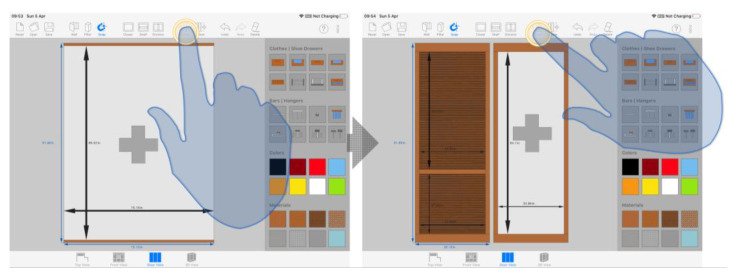
Door creation process. Each single tap on the corresponding button triggers the computations needed to parametrically define a new door and transform the position and scale of the previously created ones.

The user taps with just one finger on a GUI control to enable the corresponding tool and thus trigger the different related actions. This pattern is meant for the use of functionalities that, aimed at the creation of new objects in the scene, are implemented as automatisms and require no extra user interaction to the mere activation of the tool. As seen in Figure 5, this action pattern is evident, for example, when the user adds a new door to the design, once the external body of the cabinet has been created: when the button is single tapped, the system performs the construction of the geometric model or the new door autonomously.

##### Single Tap on a Design Element, followed by a Single Tap on a GUI Control

**Figure 6:**
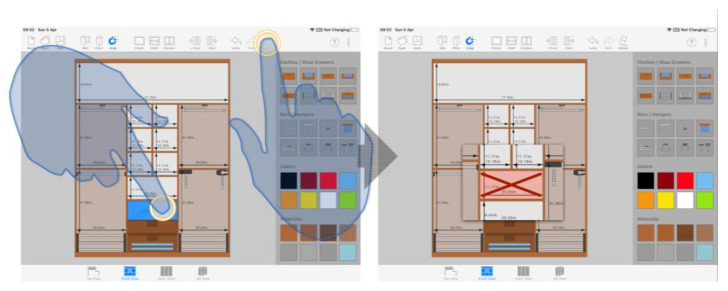
Deletion of an element from the design.

In the first place, the user single taps an object to select it. This way, the individual intentionally points at the object in the scene he wants to manipulate. Then, the subject uses his finger again to tap the button linked to the desired operation in order to modify the object previously selected. Thus, for example, to delete a drawer located in a cabinet compartment, as shown in Figure 6, the user has to select the desired drawer first and then tap on the delete button.

##### Single Tap on a GUI Control, followed by a Single Tap on a Design Element

**Figure 7:**
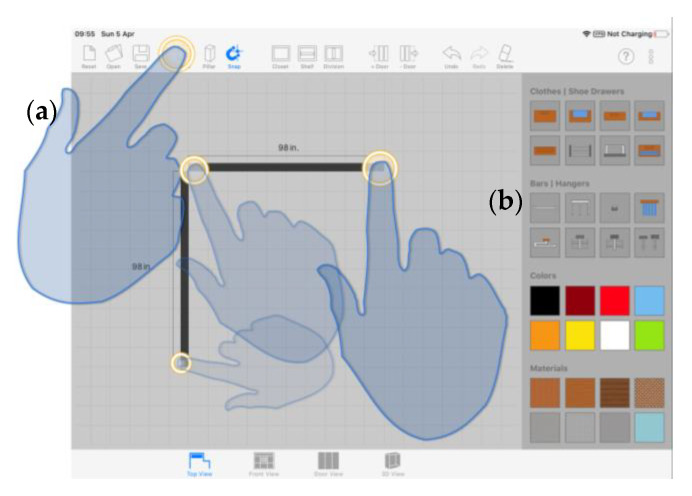
Room walls creation process: (**a**) single-tap-based wall creation tool activation and (**b**) consecutive single-taps for located element creation.

Some tools, once activated, require an extra touch on the drawing canvas to create a new object at a specific location in the scene. The user explicitly provides the location with an eminently natural gesture, pointing his finger to a particular spot. The system translates the 2D cartesian coordinates associated with the touch event into a target location for the object in the 3D space. This pattern is visible, among other tasks, in wall creation: the user first selects the tool (Figure 7a) and then creates the endpoints of the walls with successive single tap gestures (Figure 7b).

##### Single Tap on a Design Element and Drag

In addition to the object creation and deletion related tools, there is a set of functionalities that must be included in any CAD or rapid prototyping application. We are talking about object translation and scaling tools. Those operations, as we will see next, are closely related in our system.

**Figure 8:**
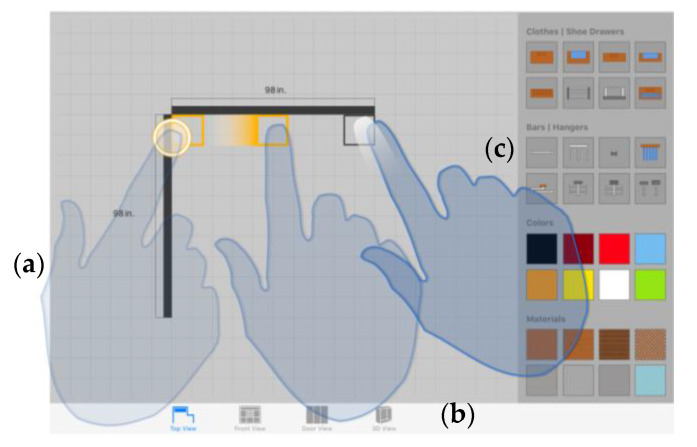
Translation of an object across the canvas: (**a**) single-tap-based object selection, (**b**) single-finger-drag-based object translation, and (**c**) finger-lift-based target object location confirmation.

To perform a translation transformation, the user must first tap the desired object to select it (Figure 8a) and then, without lifting this finger from the touch surface, drag it across the screen until the target position (Figure 8b). As the user drags the finger across the work area, the object slides in such a way that it remains under the fingertip all the time, and only when the individual lifts the finger from the touchscreen, the translation ends (Figure 8c). Such direct manipulation pattern provides the user with an engaging experience as object translation, modeled as described, is predictable and transmits to the individual the feeling of actually moving the object.

#### 3.3.2. Indirect Scaling

**Figure 9:**
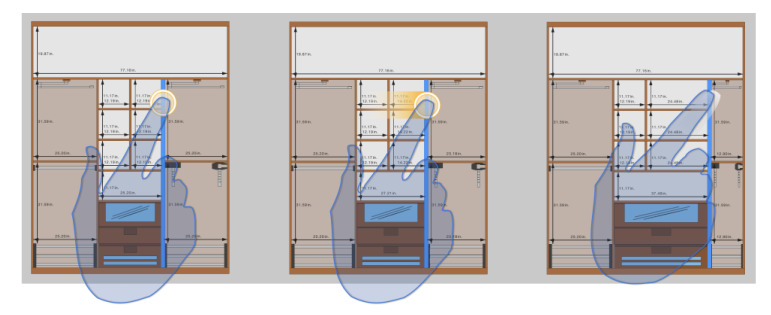
Indirect scaling application example. The algorithm modifies in real time the scale of the compartments adjacent to the horizontal division translated.

Translation does not exclusively affect the object position. Relying on this geometric transformation, we implemented an algorithm called indirect scaling that modifies the dimensions of the sketch elements connected or related to the translated object. Thus, for example, when the user moves a dividing element inside the cabinet, the system automatically resizes both the adjacent compartments and the accessories within. Figure 9 shows a graphic representation of this indirect scaling mechanism.

As can be seen, indirect scaling differs not only from the common scaling approach implemented in traditional CAD software, consisting of physical-keyboard-based direct text input, but also from the typical soft-keyboard-based approach, typical in touch systems. With respect to the latter, it was considered in the first place as the mechanism to be implemented for the sizing of the design elements due to the extensive consideration received in the related literature [51,52,53] and its widespread use in commercial multi-touch devices. However, we dropped that alternative due to its ergonomic and feedback-related problems that adversely affect users’ performance and comfort: physical keyboards provide a haptic response, allowing users to physically feel the keys as they are pressed and rest their wrists and hands while typing; in contrast, soft keyboards only provide visual feedback and prevent hands from having an ergonomically correct pose.

The finally implemented scaling mechanism enables an efficient object sizing in touch-based systems, but it is inherently less accurate than directly typing the exact values using a keyboard. To improve that, we additionally implemented a non-intrusive accuracy mechanism that does not require a change of context. Users can naturally activate the accuracy mode while sliding an object across the drawing canvas. To do that, the individual must keep the fingertip motionless on the object for 1000 milliseconds. Thereafter, a smaller scale is applied to any object translation, thus increasing the accuracy of the movement. The user can end this mode by tapping anywhere in the canvas except on the object itself.

#### 3.3.3. Mechanisms for Enhanced Target Selection

The introduced action patterns largely rely on first selecting an element of the sketch. That is common to many typical CAD tasks, which also require the selection of a specific entity or object to trigger a particular process. Whereas using a mouse and the corresponding on-screen cursor is typical in desktop CAD tools, the touch nature of our solution and the consequent absence of the cursor might force users to use fingers as pointing and selection devices, something that usually causes lack of accuracy and occlusion problems (as we pointed out in the second section of the present paper). To solve both problems, we implemented several specific mechanisms that seek to address them from different angles.

##### Additional Viewport for Displaying Relevant Information

**Figure 10:**
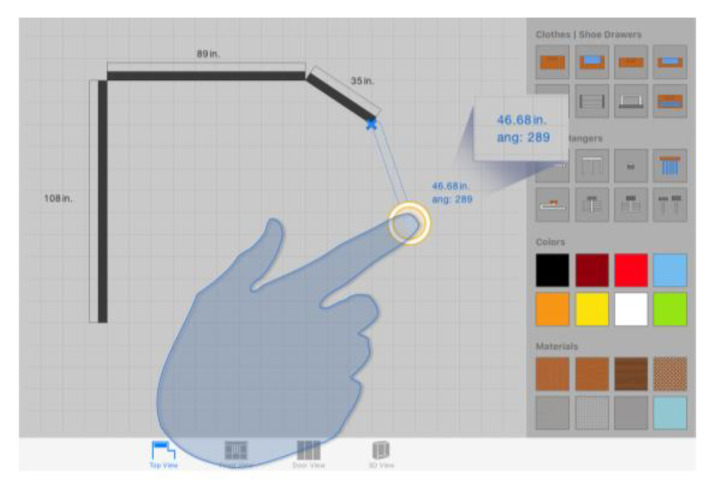
Additional viewport that provides the user in real time with the length of the wall being created.

Inspired by the Shift technique [69], this method consists also of including an additional small on-screen viewport every time a single finger taps the touch surface. However, unlike Shift, the provided viewport does not display a copy of the area occluded by the finger; it provides text information relevant in the operation context and fundamental for its successful completion when high accuracy is demanded. Figure 10 shows an example of the use of this technique to support the creation of walls.

In the depicted use case, the system provides a real-time preview of the wall as the user drags the finger across the work area. As shown in Figure 10, that “temporary” wall is rendered based on a starting point defined as the first end of the wall, and a second one defined by the position of the finger on the design slate. The finger itself prevents individuals from seeing the exact position of the second endpoint, causing the loss of an essential reference for the successful creation of the wall. In order to prevent this, the additional viewport shows the exact length of the wall with and accuracy of two decimal places.

This auxiliary display area shows up when a single finger taps anywhere in the work area, and it is located in a position close to the touchpoint. The value of the offset applied to the viewport is constant and relative to the point where the finger is touching the interaction surface. In other words, the location of the viewport is not static; it gets changed as the user drags the finger across the screen. Thanks to this, we avoid changing the user’s visual attention during the object translation. In contrast, the direction and orientation of the offset vary depending on the finger position in order to ensure that the viewport is always shown within the fixed limits of the canvas.

##### Non-Visible Expansion of the Selectable Surface of an Object

This is a mechanism inspired both by area cursors [62] and the semantic pointing technique [64], which seek to solve the accuracy problem without visually modifying the dimensions of the objects in the scene. A non-visible rectangular area is associated with each element of the sketch and acts as its selectable area: the visual information of the area is not rendered on screen but stored in an off-screen buffer. Thus, we can increase the on-screen target size to facilitate the selection task while maintaining a realistic representation of the objects.

**Figure 11:**
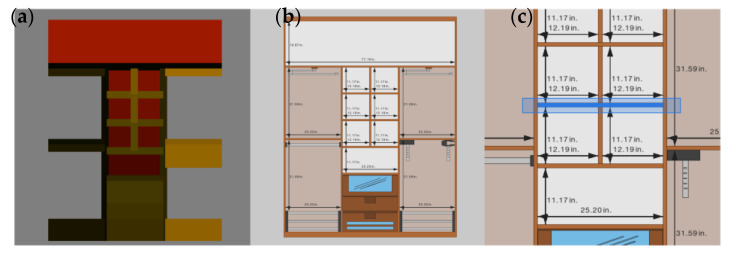
Expansion of the selectable surface of the several elements of a cabinet: (**a**) color information corresponding to the different selectable areas, (**b**) on-screen rendering, (**c**) graphical representation of the selectable surface of a shelf.

As shown in Figure 11, there is a clear center alignment between each element in the sketch and its corresponding selectable area. To expand the selectable area, we scale it up to set a new size that exceeds the dimensions of the object itself: using an x3 factor for tiny targets (up to 15 pixels per side) and an x2 factor in any other case.

Object selection is based on the color picking algorithm, common in computer-graphics-based applications. To support this algorithm, each selectable area is assigned a specific color (Figure 11a), and each object is tagged with a unique identifier consisting of the RGB color values of the corresponding selectable area. Having established that relationship, the algorithm is able to extract the color information of the specific pixel where the finger touches the selection area, and then search for the object whose identifier matches the obtained RGB data.

##### Increasing Information Space by Using Zoom

Zoom operations allow users to adjust the information space to an appropriate size for selecting on-screen targets comfortably. The implemented algorithm relies on the use of the standard multi-touch gesture known as pinch-to-zoom, to make the zoom mode activation fully transparent to the user. This mode is automatically activated when the individual starts dragging two fingers describing a pinch-in or pinch-out gesture. The zoom factor is dynamically calculated in real time from the distance between the fingers. In this sense, a finger-based pinch-out gesture will increase the zoom factor and, consequently, the display scale of the work area, while a pinch-in gesture, in contrast, will decrease both values.

The method is conceived as an alternative to the two previously introduced touch-based selection mechanisms: its use is relegated to situations where an effective selection is not possible even despite having applied both methods. More specifically, we seek to minimize the use of zooming operations by assigning them a secondary role within the overall interaction model. That is because, although gestures allow users to easily and naturally fine-tune the display size of the objects in the scene, they lead to the interruption of other tasks and consequently can hinder the design process.

### 3.4. Minimizing and Facilitating Mode Switching

As a general rule, mode switching involves a change of context that, as we have just seen for gesture-based zoom operations, usually interrupts end-user’s workflow. In this respect, it will be possible to streamline user’s tasks to the extent we are able to minimize the need to change between modes. However, the wide range of features that software design tools usually provide, hinders that minimization in a significant way: when users interact with this kind of tools, it is usual that they intentionally select the corresponding dedicated control in order to activate a new mode.

Our approach is based on two strategies that, although failing to eliminate the existence of multiple modes, do simplify the switching. These strategies consist of (*i*) providing the user with transparent access to frequently used tools and (*ii*) using multi-touch gestures to trigger mode switching. More specifically:

- The selection mode is set as the default one. This mode and the corresponding selection tool are both automatically enabled when the user ends any operation, and its associated control gets disabled.
- When the selection mode is enabled and an object is selected, a single-finger-based drag gesture enables the translation mode, as previously indicated. When the user lifts a finger from the touchscreen, the translation mode ends, and the selection mode is automatically enabled again.
- Moreover, regardless of the mode currently enabled, the system can be switched to zoom mode when the user performs a pinch gesture. As soon as the user lifts at least one finger, the zoom mode ends, and the context existing before its activation is recovered.

## 4. Evaluation

The designed interaction system, as stated in the preceding section, was implemented as part of a mobile software prototype named Sketch Arm, used as testbed for assessing the several proposed solutions we introduced. Throughout this section, we present the two usability studies conducted to test and analyze the value of the supporting interaction.

### 4.1. Remote Usability Study

As a very first approach to evaluate the usability of the multi-touch mobile prototype, we conducted an unmoderated remote study aiming to explore whether or not the adoption of a touch-based interaction model in CAD systems results in a satisfactory usability score for this type of tools; and find out to what extent the technical or professional training of users has an impact on that score.

#### 4.1.1. Participants

The mobile application itself was used as a marketing tool and incentive for the recruitment of participants, resulting in 30,699 downloads from all over the world after nine months. Out of that initial number, 1,115 individuals voluntarily took part in the study. However, contributions from subjects who either did not answer all the questions in the supplied survey or did not prove having used the tool were dismissed. Furthermore, in order to avoid potential outliers caused by the cultural diversity of the participants, we selected as final test sample merely the dataset extracted from the responses provided by Spanish-speaking individuals.

Ultimately, the sample used for the study included observations on 326 individuals, 74.8% male and 25.2% female, aged between 18 and 64 years, with 96% of them between 18 and 55 years. Out of them, more than half (61.7%) indicated not currently having or having had before any activity related to the manufacture or design of furniture. Also, the vast majority reported using tablet devices regularly and having moderate (medium or low) experience with CAD software tools.

#### 4.1.2. Procedure

Each test session took between 10 and 15 min approximately, being the followed protocol the one detailed next:

1. Introduction to the design tasks through a built-in how-to visual guide. Instructions were showcased to users when they first launched the application.
2. Creation of a new design. Participants were given the questionnaire only if they had previously created at least one sketch with the application. In this way, we ensured they had truly used the application and, therefore, we guaranteed a minimum training with the distinct considered tools.
3. Completion of the questionnaire. Once the survey had been triggered for the first time, the individual had the option of going ahead and filling it or skipping it for further completion later on. For the latter, the entire survey was presented whenever the user relaunches the application afterwards.
4. Submission of answers and comments. Once the survey was finished, all the gathered data were sent from the application, through a background submission process transparent to the user, and appropriately stored in a remote database.

#### 4.1.3. Survey

The supplied survey was designed as a four-section questionnaire:

- *Introduction of the study*. It briefly and generically describes the overall purpose of the study, and it also includes a description of the remaining sections of the survey. However, it does not provide information that could significantly influence the behavior of the participants.
- *Usability questionnaire*. It is based on the System Usability Scale (SUS) questionnaire [72], considered a standard method for measuring the usability of a given system in terms of the subjective perception of the users [73]. We have made some slight changes to its original wording in order to accommodate it to the specific context of the study, and thus facilitate its understanding. In particular, the terms cumbersome and System used in the original version of the questionnaire, were replaced by awkward [74,75] and App respectively.
- *Demographic questionnaire*. It consists of questions aimed at obtaining personal, technical and professional data from the participants: sex, age, nationality, dominant hand, whether they work or not in any activity related to the design or manufacture of furniture, how often they use of tablet devices, and the level of experience regarding the use of computer design applications. Such data allowed us to draw up a profile of the subjects that took part in the study, keeping their identity anonymous in all cases.
- *Grid view for project sharing*. Grid-based view of thumbnails corresponding to the different projects previously created with the application. In this section participants were asked to select and submit one sketch just as a sample of the level of understanding and proficiency they had achieved with the tool. As previously stated, the quality of the submitted sketch was internally used afterward as data filtering criteria in an extra step previous to analysis.

#### 4.1.4. Results

Following the procedure detailed in Brooke’s work [72], we first calculated one SUS score for each participant submission, using as input the data collected with the usability questionnaire included in the supplied survey. On the basis of those partial SUS scores, we then computed the several measures of central tendency and dispersion that characterize their distribution. Table 1 presents all the values calculated in this respect.

For the analysis of Sketch Arm’s SUS score, we interpret the calculated mean value by taking as a reference the mean SUS score and the percentile ranking provided by Sauro in [73]. We find that the calculated mean SUS score of our system (72.79) is higher than the global mean SUS score reported by Sauro (68) and that it is spotted close to the 67th percentile rank. These values give us the initial intuition that our multi-touch prototype has a usability level that exceeds the average one calculated for the several systems evaluated by Sauro. Even so, it is necessary to perform the Student’s *t*-test for one sample on the computed SUS scores to verify whether the difference we found is statistically significant or not.

We first check that the sample follows a normal distribution by performing the Shapiro-Wilk test. Since the calculated *p-value* (0.001) is below the used significance level (5%), there is sufficient evidence to reject the null hypothesis of the test (“*The sample data follow a normal distribution*”) and, consequently, to affirm that the distribution of the data is not normal. However, since the sample size is sufficiently large (326 observations >30), we can rely on the Central Limit Theorem and assume the fulfillment of the normality hypothesis.

Once the normal condition has been verified, we can perform the one-sample *t*-test. For this test, the null hypothesis (*H*_0_) assumes that the sample mean *x* does not exceed the value of the global mean SUS score used as reference *μ* (*H*_0_: *x* ≤ *μ*); on the contrary, the alternative hypothesis (*H*_1_) assumes that the value of *x* is greater than *μ* (*H*_1_: *x* > *μ*). Thus, being *x* = 72.79, *μ* = 68, *n* = 326 and *α* = 0.05, the resulting *t* statistic has a value of 6.849. For *t* = 6.849 and *df* = 325, the calculated probability is 1.86 × 10^−8^. As the *p-value* is less than 0.05, it is possible to reject the null hypothesis and, consequently, to state with nearly 100% confidence that our system has obtained a mean SUS score higher than the industry average of 68.

To determine whether subjects’ technical and professional skills affect the final SUS score computed for Sketch Arm, we perform a factorial ANOVA (an Analysis of Variance test with more than one independent variable) on the dataset of previously calculated partial SUS scores. More specifically, we opt for a 2 × 3 × 3 factorial design in which:

- Three factors are considered: the subjects’ level of experience with CAD tools (CAD_Exp), how often they use tablet devices (USE_Freq), and whether they have performed or not professional activities related to furniture manufacturing or design (IS_Prof).
- Both the CAD_Exp and USE_Freq factors have three distinct levels (High, Medium and Low for the CAD_Exp factor; Strong, Frequent and Sporadic for the USE_Freq factor) while the IS_Prof factor has two levels (Yes, No).

Based on the factorial ANOVA, we evaluate both individual and combined effects of the three independent variables on the SUS score, analyzing whether or not there are significant differences between the mean scores calculated for each group. Again, as a desirable previous step, we check the normal condition in every considered population group, but we also check whether those groups present or not the same variance. Since the design contemplates eighteen different population groups, to do so, we perform the Shapiro-Wilk test (normality testing) and Levene’s test of homogeneity of variance (homoscedasticity testing) on residuals (that is, the difference between observed and predicted values) in order to perform both tests simultaneously for all the studied groups.

The Shapiro-Wilk test provides a *p-value* of 0.061, higher than the 0.05 significance level. Consequently, it is not possible to reject the normality hypothesis, so we can assume that the residuals follow a normal distribution. For its part, with regard to Levene’s test, the resulting values obtained are 0.730 for the test statistic and 0.744 for the *p-value*. Therefore, the null hypothesis (“*population variances are equal*”) cannot be rejected, so we can conclude that residuals meet the homoscedasticity condition and also that the variances of the several considered groups are homogeneous.

Having tested compliance with normality and homoscedasticity assumptions, we are ready to perform the factorial ANOVA test. We set null hypothesis both for each individual factor and for each possible factor combination. More specifically, as factor-specific null hypothesis we assume that the mean values corresponding to the different populations defined by the several levels of the factor are equal, while as combination-specific null hypothesis we assume that the interaction of the factors has no effect. We test all the considered hypothesis by calculating the *F* statistic and the associated critical level, being the latter, the reference value used later on to decide whether we reject or not each hypothesis. The following table presents the set of values of the statistic and its corresponding *p-value* obtained for the several considered factors and factor combinations.

We see in Table 2 that every *p-value* calculated for each particular factor is higher than 0.05. Thus, we cannot reject the hypothesis of equality of means, so we can conclude that the SUS scores of the different groups defined by each factor are virtually equal. Similarly, with regard to the effect of the factor combinations, the critical level obtained, for nearly all of them, is higher than the considered significance value. Only *CAD_Exp* and *IS_Prof* interaction reports a significant effect on the SUS score (*p-value* = 0.024).

Regarding the latter, to get a deeper insight about which specific group the discovered difference lies in, we compare each group with the considered others by performing a *post hoc* multiple comparison test. As result, we find that the difference between the average SUS scores obtained for professional and non-professional users is only statistically significant within the population group of individuals with *Medium* CAD-related experience (*p-value* = 0.017). Likewise, *post hoc* comparisons between *CAD_Exp* levels within each *IS_Prof* level reveal that (*i*) within the group of non-professional participants the average SUS score computed for individuals with a *High* level of experience is significantly different from the one obtained for subjects with both *Medium* (*p-value* = 0.009) and *Low* experience (*p-value* = 0.010); and that (*ii*) within the group of professional users the difference is significant only between participants with a *High* experience level and those with a *Low* one (*p-value* = 0.022).

### 4.2. In-lab Comparative Usability Study

Besides the remote testing detailed, an additional study was conducted, conceived as a supervised in-lab A/B test. In particular, we compared our multi-touch interaction model against a more traditional WIMP-based alternative. To do so, we used as a benchmark not only the user’s satisfaction (expressed in terms of SUS score), but also several observable and measurable performance parameters, extracted directly from the official definition of usability provided in ISO 9241-11 [76]. More specifically, the attributes additionally considered were effectiveness and efficiency, being the corresponding representative metrics the following ones:

- Time. Metric referred to how much time a subject spends in each test scenario. That time value was measured in seconds, expressed with three decimal places, and it was quantified as the time elapsed from the moment the system signals the beginning of a task to the moment the subject clicks on the button intended for finishing it.
- Error rate. Metric that represents the number of unsuccessful operations out of the total number of actions performed by the subject in each test scenario. Clicking on the Undo button, along with any failed selection and positioning of the elements in the design, were considered wrong actions.

#### 4.2.1. Participants

A total of 42 subjects took part in the study’s test sessions on a *pro bono* basis. The group consisted exclusively of computer science college students, 22 males and 20 females between 19 and 25 years old. Given the degree in which they were enrolled and the consequent specific IT training previously received, all the participants had excellent computer skills and were also daily users of multi-touch devices such as smartphones and tablets. Still, none of them claimed to have previously used design software tools similar to Sketch Arm or to have been involved in any activity related to furniture design or manufacturing.

#### 4.2.2. Software Tools and Apparatus

**Figure 12:**
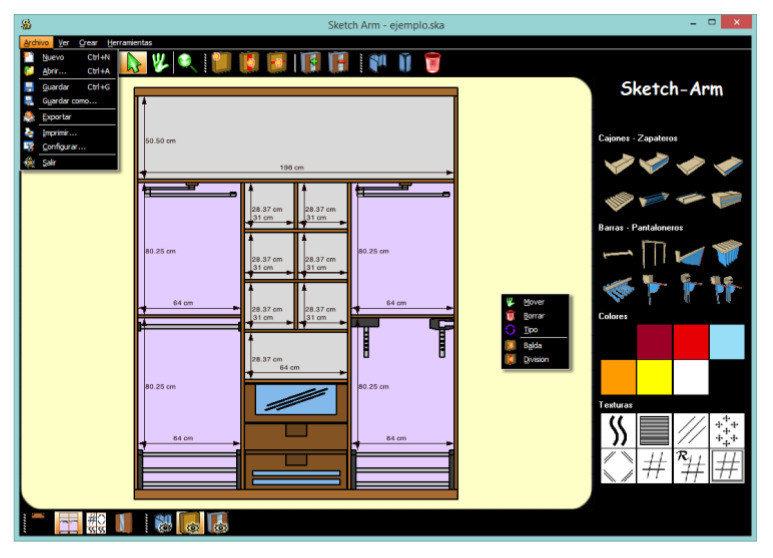
Look and feel of Sketch Arm’s GUI for desktop systems.

A WIMP-based alternative to the touch-based interaction model was developed as a functional desktop prototype supported by mouse-based data entry and an old-fashioned GUI. For the GUI, in particular, we created a layout nearly identical to the one previously described for the touch-based system, with a single window (Figure 12) containing all the controls necessary to perform the different operations involved in the cabinet design process, but also with two additional traditional desktop-specific elements that facilitate access to the on-screen controls: a menu bar and a context menu.

Test sessions, including the design tasks performed using both the desktop prototype and Sketch Arm, were recorded on video, capturing every subject’s on-screen activity in real time. As recording equipment, we used a laptop with a 13-inch screen, a resolution of 2560 × 1600 pixels and a pixel density of 225 pixels per inch (ppi). The same computer was used by the participants to carry out tasks with the desktop prototype, whereas tasks with the multi-touch solution were performed using a third-generation iPad, a tablet device with a 9.7-inch capacitive multi-touch screen, a resolution of 2048 × 1536 pixels and a pixel density of 264 ppi.

Along with the described hardware, we used a screen recording application called Silverback. The software, which could be exclusively run on desktop systems and thus just on the laptop used in the test sessions, strictly speaking, allowed only the recording of the interaction of the user with the desktop prototype. To do the same with the multi-touch prototype, it was necessary to use an additional software tool called Reflector, a comprehensive mirroring solution for mobile devices. Thanks to that application, it was possible to capture the participants’ on-screen real-time activity with the tablet device and send it to the connected recording machine. Using that software together with Silverback simplified the recording process, avoiding complex setups, typical in usability testing of mobile systems.

#### 4.2.3. Design and Experimental Procedure

The conducted A/B test was based on a within-subject design with repeated measurements. Two independent variables were considered for the experiment: the used *Prototype* (*Desktop* and *Multi-touch*) and the several *Tasks* performed in every test cycle (*T*1, *T*2, and *T*3). Each combination of both variables was tested by 42 subjects. Thus, in short, the design resulted in a total of 42 participants × 2 Prototypes × 3 Tasks = 252 trials.

Each test session was attended by just two participants simultaneously, each one accompanied by a researcher. The duration of those sessions was approximately between 35 and 45 min. That time frame was distributed among the several steps of the applied protocol as follows: (*i*) introduction and welcome, 10 min; (*ii*) informed consent signing, 5 min; (*iii*) tasks and prototypes introduction, 6 min; (*iv*) design tasks, 10-20 min; and (*v*) delivery of rewards and farewell, 5 min.

All the participants performed each proposed task twice, once for each prototype. To prevent a possible bias or preference towards any of the tested prototypes, we used a crossover design, randomly assigning each subject the prototype order use. Regardless of the order, the purpose of the tasks was providing participants with a real-work-like framework representative of the cabinet design process. For this reason, a total of three tasks were proposed: *T*1 related to room blueprint sketching, *T*2 corresponding to the interior layout editing module, and *T*3 related to the door editing module. *T*1 and *T*3 tasks were based on a shared underlying dynamic, the on-screen rendering of a reference model that the participant had to replicate as accurately and efficiently as possible by mainly performing selection, scaling and rotation operations. *T*2, for its part, was conceived from a slightly different approach, enabling individuals to have total creative freedom to propose a possible cabinet interior design, given the dimensions of the cabinet but based on their specific real-life storage needs. Moreover, once the task cycle was completed, each participant was also asked to evaluate their experience with the prototypes by filling out the corresponding SUS questionnaire.

#### 4.2.4. Results

To determine which of the two compared designs had better usability in terms of performance (time and error rate) and user satisfaction, we perform several paired sample *t*-tests on the gathered data. That *t*-test assumes as null hypothesis that the mean difference between paired observations is zero, and, like any other parametric procedure, it previously demands checking the normal condition on the differences between pairs to guarantee its power. For both *t*-tests and normality tests, the used level of significance (α) is 0.05.

##### Overall Performance of Prototypes

The computed average task completion time has been very similar for each prototype: 3.096 min (σ = 0.937) for the *Multi-touch* prototype and 3.466 min (σ = 0.796) for the *Desktop* prototype. The Shapiro-Wilk test performed on the time differences (*W* = 0.948, *p-value* = 0.082) results in a *p-value* higher than α. Consequently, the null hypothesis can be rejected, and we can assume that the distribution of the differences is normal. With the assumption of normality fulfilled, the results provided by the paired *t*-test (*t* = 3.562, *p-value* = 0.001) finally show a statistically significant difference between the mean values obtained for the time associated with each prototype (*p-value* < 0.05).

In addition, with regard to the overall average error rate calculated, the value finally obtained for the *Desktop* prototype is 12.72% (σ = 4.35), while the one obtained for the *Multi-touch* prototype is 20.14% (σ = 7.15). Assuming the normality of differences, supported by the results of the Shapiro-Wilk test (*W* = 0.979, *p-value* = 0.635), the *t* statistic (−6.211) and *p-value* (2.39 × 10^−7^) resulting from the *t*-test reveal an existing significant difference between the mean value of the error rates computed for each prototype (*p-value* < 0.05).

##### Task-Specific Performance of Prototypes

**Figure 13:**
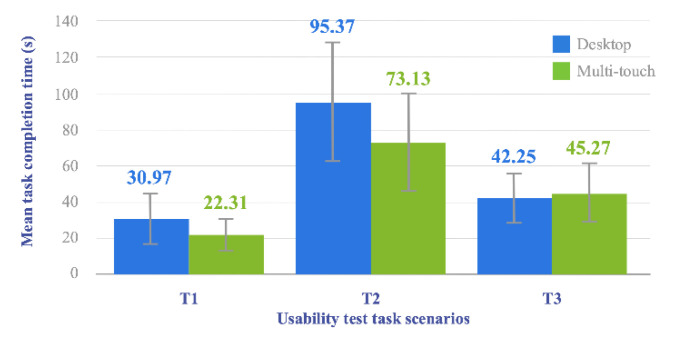
Mean completion time values for T1, T2 and T3 tasks performed both with Desktop and Multi-touch prototypes.

The mean task completion times (in seconds) obtained for the different considered *Prototypes* and *Tasks* can be consulted in the chart shown in Figure 13. The figure depicts the extent to which the *Prototype*, and therefore the UI, affects the amount of time required by individuals to perform the different CAD-related *Tasks* conceived as test scenarios. Specifically, the bar chart presents the mean task completion time per *Prototype* clustered by *Task* but also provides a visual representation of the standard deviation of each sample in the form of an error bar. Leaving aside the specific numerical values, such standard deviation error bars and their overlapping level give preliminary intuition on whether or not there is a significant difference in terms of task completion time between the two *Prototypes* under consideration. In particular, in Figure 13, with a mere visual inspection, it is possible to observe a clear overlap between the error bars corresponding to the two tested solutions. Therefore, it can be presumed that the difference between them is not statistically significant.

As shown in Table 3, the Shapiro-Wilk test performed on the differences in task completion time results in a much higher *p-value* than the *α* value considered. We, therefore, assume the condition of normality in the three corresponding samples. Furthermore, results obtained from the paired *t*-test, show that there is a difference statistically significant between the mean time values computed for each prototype only in T1 and T2 tasks (*p-value* < 0.05).

**Figure 14:**
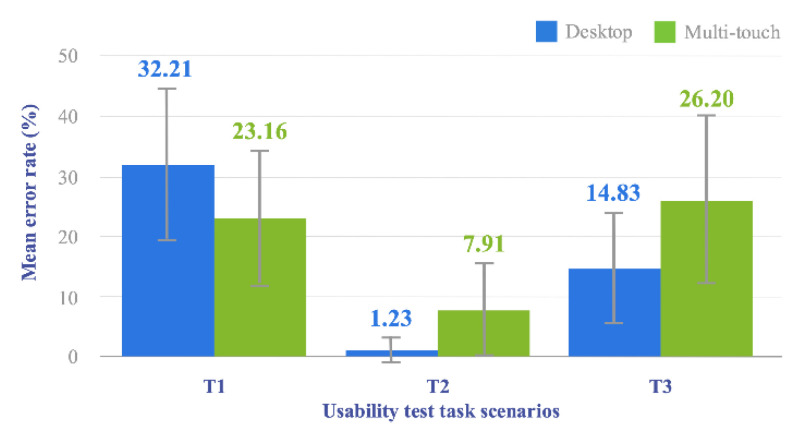
Mean error rate values for T1, T2 and T3 tasks performed both with Desktop and Multi-touch prototypes.

Likewise, with regard to the error rate (expressed as a percentage) derived from the participants’ activity, results obtained for the *Multi-touch* system are, except for the results related to task *T*1, higher than the ones obtained for the *Desktop* system (Figure 14). Moreover, as with the plot in Figure 13, the overlap between the standard deviation error bars of both systems is noticeable in all the tasks, pointing to a potentially non-statistically significant difference between their corresponding error rate values. Such error bars are indeed considerably long in this case, revealing a higher data variability and, consequently, greater uncertainty in the error measurements reported.

To test the normal condition, for each *Task*, we compute the difference between the error rate of each prototype. Supported by the results provided by the Shapiro-Wilk test (Table 4), we can assume the normality of the data. The *p-value* obtained after testing the three samples of calculated differences between error rates, is higher than the α value considered in all cases. It is, therefore, possible to accept the null hypothesis and assume that the three samples come from a normal population. Finally, the results of the paired *t*-test show the existence of a statistically significant difference between the error rates obtained for the *Desktop* and *Multi-touch Prototypes* in the three *Tasks* performed by individuals during the test sessions. 

##### Subjective User Evaluation

The overall mean SUS score obtained for the *Desktop* prototype is 78.72 (σ = 11.89), while the one for the *Multi-touch* prototype is 80.06 (σ = 11.70). The results provided by the Shapiro-Wilk test (*W* = 0.945, *p-value* = 0.058) allow us to assume the normality of the differences between the paired SUS scores of the compared devices. Lastly, the paired sample *t*-test performed on the two samples of SUS scores computed for each prototype (*t* = −0.762, *p-value* = 0.451) shows that the difference between the corresponding mean scores is not statistically significant (*p-value* > 0.05). 

## 5. Discussion

Supported by Sketch Arm, a multi-touch software prototype for modeling custom cabinets, we conducted two user studies to assess the touch-based CAD user interface conceived in this work. As a first effort to analyze and evaluate the usability of the proposed interaction mechanisms, a non-moderated remote study was conducted with the aim of (*i*) gaining a preliminary sense of the resulting interface’s usability and (*ii*) finding out the effect of the users’ expertise on usability results. Supplementing that massive study, further experimentation with users was conducted, in this case, in a laboratory. Specifically, the multi-touch approach was compared with its desktop twin not only in terms of SUS score but also taking into account performance metrics such as the time spent and the errors made by the participants when performing CAD tasks.

In terms of design tasks, the multi-touch prototype allowed participants to complete trials in significantly less time (*time* (*Multi-touch*) = 3.096 min) than the time spent using a desktop system (*time* (*Desktop*) = 3.466 min). Such gain was obtained potentially thanks to the more direct nature of the touch interface, compared to a traditional mouse-based interaction alternative. As noted at the very beginning of the section regarding the proposed selection mechanisms, the several action patterns conceived to address the design process of custom cabinets are firmly based on selection operations. In that sense, it is possible to state intuitively that such operations are performed intensively for the type of operations considered and that, by extension, they have a significant impact on the overall interaction. In that context, it has been possible to streamline interaction by reducing selection time: touch interaction allows users to direct select and manipulate any on-screen element; on the contrary, with desktop systems, users have to move the mouse pointer to the target position before confirming the selection.

For its part, results obtained in relation to the number of errors committed by individuals during test sessions undoubtedly point to a better performance of the traditional-interaction-based system. Our work has primarily been focused on solving, or at least minimizing, some of the main drawbacks of finger-based touch interaction, succeeding, as we have just seen, in reducing interaction time; unfortunately, it has not achieved the same for the error rate. Using a point-based graphic cursor operated by a mouse helps users to perform pointing and selection related tasks with a higher accuracy level, mainly when on-screen targets are small-sized, as it is the case with some of the typical elements of a cabinet sketch such as shelves or walls.

Moreover, task-based-clustered completion time results, depicted in Figure 13, allow a deeper understanding of user performance on creative duties like CAD-specific tasks. Though it is not possible to clearly identify a trend or pattern among the reported outcome measures, it is evident that time values obtained for *T*2 task, both with the *Desktop* and the *Multi-touch* prototypes, are considerably higher than the ones obtained for the two other tasks. *T*2 has been, therefore, the most time-consuming task (*time* (*Multi-touch*) = 95.37 s, *time* (*Desktop*) = 73.13 s) and, consequently, it is reasonable to think as well that it has been the most demanding of the three conceived for the conducted in-lab experimentation. Also, given the participants’ non-professional profile, it might seem appropriate to attribute such high time values to the lack of CAD-specific expert knowledge typical of mainstream users. However, a comparison with the times reported for the remaining two tasks invalidates that hypothesis as it reveals substantially lower values for both *T*1 (*time* (*Multi-touch*) = 22.31 s, *time* (*Desktop*) = 30.97 s) and *T*3 (*time* (*Multi-touch*) = 45.27 s, *time (Desktop)* = 42.25 s) while requiring the aforementioned CAD expertise too. It is by focusing purely on design aspects that one can notice some difference between those two tasks and *T*2 that might shed some light on the high completion time associated with the latter. In particular, as mentioned in Section 4.2.3, only in *T*2 task participants had absolute freedom regarding design operations. In that sense, it can be seen as a creative process involving not just mere sketching or modeling, but also a prior preparation stage [77] in which individuals must face the so-called blank canvas paralysis [78] by taking additional time to analyze the problem being addressed and devise possible solutions.

Finally, results regarding user satisfaction have been encouraging. Even though the average SUS score obtained by the *Multi-touch* prototype was higher than the one computed for the *Desktop* alternative, that difference was not statistically significant. Thus, although our multi-touch interaction system showed a higher user satisfaction, we cannot extend that result to the entire population, and it should be considered a mere random occurrence. Even so, regardless of the statistical significance of the difference, it is worth emphasizing the high mean SUS score obtained for the two tested prototypes. The reported resulting values suggest that, regardless of the input mechanism used, the several action patterns proposed to address cabinet-specific design tasks are intuitive and make the resulting systems very attractive and easy to use.

If we go into more detail and we take as an additional reference the remote study conducted in the first place, we can also see that factors such as previous experience with CAD tools (*CAD_Exp*), proficiency using multi-touch mobile devices (*USE_Freq*) or field-specific professional skills and experience (*IS_Prof*), contrary to what we might think beforehand, did not impact on subjects’ perceived usability of the multi-touch system. Only the interaction of *CAD_Exp* and *IS_Prof* have shown evidence of having impacted on the scores provided by the participants, being the population sample consisting of non-professional individuals with CAD-related previous experience the one that has provided the most positive response regarding the multi-touch prototype and, therefore, regarding the interaction model proposed.

## 6. Conclusions

CAD software makes it possible to deal with rapid prototyping and 3D modeling tasks, both required for the conception of new products in AM. However, the penetration of those tools beyond the professional field remains scarce or non-existent due to their technical complexity and the demand for expert knowledge that mainstream users do not usually possess. In this work, we have designed a novel touch UI that, supported by current multi-touch hardware, integrates several interaction solutions that seek to facilitate low-IT-skilled users the operation of CAD tools towards a new and optimized AM model in which the final customer is responsible for the design of the products to be manufactured.

The human-machine communication during the creative process approached using CAD tools, has been modeled as a set of design supporting interactions characterized in the form of action patterns: (*i*) single or multiple taps on a GUI control to add new elements to the design; (*ii*) single tap on a design element, followed by a single tap on a GUI control to perform a specific operation on a particular object; (*iii*) single tap on a GUI control, followed by a single tap on a design element, to include a new element in the scene as well, but in a specific location; and (*iv*) single tap on a design element and drag to move and scale the objects in the scene.

Besides, concerning scaling operations, the paper presents two additional contributions: a new algorithm named as “indirect scaling” that makes it possible to modify the size of the elements present in design straight away with the finger, bypassing the need to introduce specific measurements; and a non-intrusive mechanism for improved accuracy of scaling operations, based on a “pseudo-mode” enabled when the user momentarily holds his finger on the object to be moved.

Knowing the occlusion and lack of accuracy problems typical in touch handheld devices operated with the bare hand, several specific techniques have also been conceived to improve finger-based selection operations:

- Additional viewport for displaying relevant information, intended for the temporary display of information concerning the operation performed in an unobscured screen area.
- Non-visible expansion of the selectable surface of an object, which involves the scale up of objects only in motor space, thus facilitating their selection with no need to affect their visual appearance and, therefore, the user’s perception.
- Increasing information space by using zoom, which allows the user to set a visual scale better suited for comfortable selection by means of industry-standard pinch gestures.

The abovementioned contributions, as evidenced by the results arising from the different user studies conducted, have succeeded in improving user performance when performing CAD-related tasks, as well as providing a high level of satisfaction, which certainly illustrates the feasibility of the targeted customer-center HMI for AM. However, experiments have also revealed a substantial error rate. In that sense, in future work, it would be worthwhile to take up again the accuracy-related problem of touch systems and explore alternative approaches for a more effective interaction, such as providing haptic or auditory feedback to the user in response to touch-based input or going further with the use of symbolic gestures to get rid of GUI controls and, thus, conceive a new interface as a pure design slate. Furthermore, since in CAD software it is not unusual to work with small-sized elements like points or polylines, it would be desirable to explore possible techniques for the effective selection and manipulation of such elements in touch contexts, by adapting, as necessary, the mechanisms already proposed to that type of objects, or even by designing new specific solutions.

Finally, although conceived interaction solutions have focused on simplifying design tasks by enhancing the usability of supporting CAD tools, experimentation results suggest a potential additional complexity of design tasks simply because of their mere creative nature. As indicated in the previous section, the creative process begins with an eminently negative stage in which the subject can be flooded with feelings such as anguish, despondency, or insecurity [77] in the absence of ideas. For the transition to a customer-centric AM model it will be necessary to overcome that early barrier that prevents the user from being totally satisfied and that, in many cases, can lead to customer loss. With that in mind, by incorporating decision-making support algorithmic solutions such as the one presented in [19], it will be possible to move towards a new HMI concept that may autonomously provide design proposals and thus constitute a framework to facilitate the user the conceptions of new ideas. In that sense, it will be necessary to explore alternatives to turn the HMI into an expert system, as well as mechanisms for their empirical evaluation.

## Figures and Tables

**Table 1:** Main measures of the central tendency and dispersion of the distribution described by the computed partial SUS scores.

Central Tendency		Dispersion	
Mean	72.79	Standard deviation	12.63	
Median	72.50	Minimum	42.50	
Mode	70	Maximum	100	

**Table 2:** *F* statistic and critical level values for the several considered factors and their possible combinations.

Factor	F	*p*-Value
CAD_Exp	2.138	0.120
USE_Freq	0.034	0.967
IS_Prof	0.043	0.836
CAD_Exp*USE_Freq	0.248	0.911
CAD_Exp*IS_Prof	3.782	0.024
USE_Freq*IS_Prof	0.563	0.453
CAD_Exp*USE_Freq*IS_Prof	0.944	0.390

**Table 3:** Results from the Shapiro-Wilk test performed on the computed time differences, and from the *t*-test performed on the paired time samples, for each task considered in the in-lab usability study.

	Shapiro-Wilk Test		Paired *t*-Test	
	W	*p*-Value	t	*p*-Value
*T1*	0.978	0.120	3.718	6.46 × 10^−4^
*T2*	0.988	0.933	4.274	1.2 × 10^−4^
*T3*	0.987	0.926	−1.147	0.258

**Table 4:** Results from the Shapiro-Wilk test performed on the computed error-rate differences, and from the *t*-test performed on the paired error-rate samples, for each task considered in the in-lab usability study.

	Shapiro-Wilk Test		Paired *t*-Test	
	W	*p*-Value	t	*p*-Value
T1	0.962	0.181	3.272	2.2 × 10^−3^
T2	0.953	0.103	−5.387	4 × 10^−6^
T3	0.976	0.564	−4.631	4.2 × 10^−5^
